# Association Between Socioeconomic Status and Emergency Department Use for Non-traumatic Dental Conditions

**DOI:** 10.5811/westjem.47316

**Published:** 2026-01-21

**Authors:** Heather Taylor, Paul Musey, Adam Hirsh, Thankam Thyvalikakath, Joshua R. Vest

**Affiliations:** * Indiana University Richard M. Fairbanks of Public Health, Department of Health Policy and Management, Indianapolis, Indiana; † Indiana University School of Medicine, Department of Emergency Medicine, Indianapolis, Indiana; ‡ Indiana University School of Science, Department of Psychology, Indianapolis, Indiana; § Regenstrief Institute, Center for Biomedical Informatics, Indianapolis, Indiana; || Indiana University School of Dentistry, Department of Dental Public Health & Dental Informatics, Indianapolis, Indiana

## Abstract

**Introduction:**

Among ED visits, presentation for a non-traumatic dental condition represents one of the most preventable, as 79% of these visits are considered avoidable. Our goal was to investigate the association between individual-level socioeconomic status (SES) and emergency department (ED) use for non-traumatic dental conditions.

**Methods:**

In this retrospective, pooled cross-sectional analysis we used data from a database of administrative health claims for members of large commercial and Medicare Advantage health plans. The sample included adults (≥ 18) who presented to the ED between 2017–2021 and had complete data on SES indicators (ie, income, education level, net worth, homeownership, and low-income subsidy status). The primary outcome was ED use for non-traumatic dental conditions, identified via International Classification of Diseases diagnosis codes. We used multivariate logistic regression models with marginal effects to examine the relationship between SES and ED visits, adjusted for demographics, geographic region, and disease burden.

**Results:**

Among 3,894,785 individuals, 74,685 (1.9%) had an ED visit related to non-traumatic dental conditions. Lower SES was significantly associated with increased ED visits for these conditions, with income exhibiting the strongest effect. Compared to individuals earning > $100,000 annually, those earning < $40,000 were 0.7 percentage points (1.5% vs 2.2%) more likely to visit the ED for non-traumatic dental conditions (P < .001). A dose-dependent effect was observed for the composite SES score, with individuals in the lowest SES quartile 1.3 percentage points (1.3% vs 2.6%) more likely to visit the ED compared to the highest quartile (P < .001).

**Conclusion:**

Lower socioeconomic status is associated with higher ED use for non-traumatic dental conditions, underscoring disparities in oral healthcare access. Targeted policy interventions and better integration of oral and medical healthcare systems are needed to reduce preventable ED visits.

## INTRODUCTION

Socioeconomic status (SES) reflects an individual’s access to resources such as goods, money, social networks, healthcare, and education, all of which influence the ability to prosper and thrive.^1^ Because access to these resources also affects one’s capacity to manage stress, afford healthcare, and make healthy choices, SES has a greater influence on health outcomes than clinical care or health behaviors.^2^–^5^ Those with low SES not only experience higher rates of morbidity and mortality than their high SES counterparts, but are also 2.5 times more likely to visit the emergency department (ED) for preventable reasons.^6^–^9^ Given the high costs associated with preventable ED visits, understanding individual SES is particularly useful for health systems that wish to reduce avoidable ED visits, minimize costs, and identify vulnerable populations.

Among the types of ED visits, non-traumatic dental conditions represent one of the most preventable, as 79% of these visits are considered avoidable.^10^,^11^ Because EDs are not equipped to provide definitive dental care, patients often receive suboptimal treatment, contributing to clinicians’ frustration, crowded EDs, and elevated healthcare costs.^11^–^17^ Adults who are socially vulnerable disproportionately drive these visits, reflecting broader issues of poor oral health among underserved populations.^18^–^20^ The reliance on EDs for treatment of non-traumatic dental conditions among these individuals is multifaceted, stemming from the intersection of unmet dental needs and socioeconomic challenges. For example, adults with low SES face higher rates of oral diseases (such as caries, periodontal disease, and oral cancer) compared to those with higher SES.^21^–^25^ These conditions are exacerbated and frequently go untreated due to barriers like limited access to dental care and underuse of preventive services among low-SES groups.^19^,^26^…Despite these clear connections, research has yet to empirically examine how individual-level SES factors—such as income, education, and housing—influence ED use for non-traumatic dental conditions. Instead, prior studies have primarily focused on broader social determinants of health, such as community-level poverty and median income, leaving critical individual-level SES factors unexplored.^13^,^27^–^35^

To address this gap in the literature, this study investigates the association between an individual’s SES and preventable ED use for non-traumatic dental conditions. Using national claims data enriched with patient-level socioeconomic indicators—including education-level, income-level, net worth, and homeownership—we examine how SES-related factors are associated with ED use for non-emergent dental care. Given that SES represents access to several resources, we also examine how a composite score of SES is related to ED use for non-traumatic dental conditions. Our analysis also accounts for individual-level demographics and disease burden, acknowledging the established relationship between poor oral health and age, race, sex, and chronic diseases. 19,36–39 By shedding light on the role of individual-level SES1 in this study we aim to help inform clinicians and healthcare administrators in developing strategies to reduce avoidable ED visits for non-traumatic dental conditions. The findings are also valuable to health systems and payors that are increasingly collecting health-related social need factors to more accurately model outcomes and strategically allocate resources in the ED. Finally, this study demonstrates the value of leveraging health and social data to better understand and identify oral health disparities.

**Population Health Research Capsule**

What do we already know about this issue?

*Lower socioeconomic status is linked to poorer oral health and higher use of emergency departments (ED) for preventable conditions*.

What was the research question?

*Are individual-level socioeconomic factors associated with ED visits for nontraumatic dental conditions?*

What was the major finding of the study?

*The lowest socioeconomic quartile was 1.3% more likely (2.6% vs. 1.3%, 95% CI, 1.3–1.3; P < .001) to visit the ED for nontraumatic dental conditions*.

How does this improve population health?

*Findings highlight how socioeconomic disparities are associated with preventable dental ED visits and inform policies to improve oral healthcare access*.

## METHODS

The study adhered to the Strengthening the Reporting of Observational Studies in Epidemiology (STROBE) reporting guidelines^39^ and was deemed exempt by the Indiana University Institutional Review Board (No. 2011541510) because it did not involve human subjects as defined in 45 CFR 46.102(f).

### Study Design and Data Source

We conducted a retrospective pooled cross-sectional analysis study using data derived from Optum’s Clininformatics® Data Mart (CDM). The database comprises administrative health claims from a large, national, managed care company and Medicare Advantage health plan members.^40^ It contains de-identified health claims for members (who reside in all 50 states) with both medical and prescription drug coverage. We restricted our analysis to continuously enrolled adults (≥ 18 years of age) who had a minimum of 12 months of coverage and who had visited an ED between the calendar years 2017–2021 (N = 7,613,265). In addition to administrative claims data, Optum’s CDM contains a separate dataset (“CDM SES data”) with information on member’s socioeconomic characteristics, including home ownership, net worth, income level, low-income subsidy status, and education level. These data were linked to each member identified as having an ED visit during the study time frame using Optum’s synthetic patient identifiers.

### Measurements

#### Outcome

For the main dependent variable, we generated a binary indicator as to whether the adult had any ED visit related to a non-traumatic dental condition during the study timeframe. To determine whether an ED visit was related to a non-traumatic dental condition, we examined all diagnoses codes reported in each individual ED claim. If the claim contained one of the *International Classification of Diseases, 10^th^ Revision* (*ICD-10*) diagnoses codes indicative of non-traumatic dental conditions (see Appendix), then this visit was considered an ED visit related to a non-traumatic dental condition.^41^

#### Individual Socioeconomic Indicators

For each individual included in this study, we extracted the following indicators of SES: education level (high school degree or less, less than a bachelors’ degree, bachelors degree of higher); home ownership status (homeowner, renter); income level (< $40,000; $40,000–$49,000; $50,000–$59,000; $60,000–$74,000; $75,000–$99,000; and ≥ $100,000); net worth (< $25,000 $25,000–$149,000; $150,000–$249,000, $250,000–$499,000, ≥ $500,000); and low-income subsidy status (ie, whether the individual was dually eligible for Medicaid and Medicare) (yes, no). To generate a composite SES score, we encoded each SES indicator into numeric values. For example, education levels were assigned values such that “high school degree or less” was coded as 1, “less than a bachelor’s degree” as 2, and “bachelor’s degree or higher” as 3. Similarly, the lowest category of each indicator was encoded as 1, with subsequent categories assigned incrementally higher values.

To ensure equal weighting of each SES indicator in the composite score, the numeric values were normalized by dividing each observed value by the maximum possible value for that variable. This normalization scaled all SES indicators to a consistent range between 0–1, where the highest category of each indicator was assigned a normalized value of 1. For each individual, the normalized values of all SES indicators were summed to calculate the composite SES score. This approach ensured that each indicator contributed equally to the total SES score, regardless of its original number of categories. Finally, we transformed the composite SES score into a categorical variable by grouping it into quartiles, with the highest quartile representing those with higher SES.

#### Independent Variables

We included individual-level demographic factors such as race and ethnicity (Asian, Black, Hispanic, White), sex (female, male), age (18–34 years of age, 35–54, 55–64, 65–74, 75–84, and ≥ 85 years of age), and geographic census region of residence (New England, Middle Atlantic, East North Central, West North Central, South Atlantic, East South Central, West South Central, Mountain, and Pacific). To capture each individual’s overall disease burden, we used the Elixhauser Comorbidity Index (ECI) to quantify each individual’s burden of comorbid conditions. To control for an individual’s overall level of healthcare use, we calculated the total number of ED visits for all individuals across the five-year study period. We also calculated the number of years each individual was represented in our analytic dataset for use as a control variable.

### Analysis

We restricted analyses to those individuals who had no missing values (n = 3,894,785; 52.2%) for any of the SES indicators. We employed descriptive statistics, including frequencies and means, to characterize the demographic, socioeconomic, and disease burden profiles of the study sample. Bivariate relationships between ED use for non-traumatic dental conditions and demographic, socioeconomic, and disease burden factors were analyzed using chi-square tests. Because odds ratios cannot be compared across different population samples or interpreted as absolute effects,^43^ we chose to estimate marginal effects of each SES indicator—education, income, net worth, and homeownership—adjusted for all demographic and disease burden factors included in the multivariate logistic regression model. Additionally, we estimated the marginal effects of each individual’s composite SES score (categorized) while accounting for demographic and disease burden characteristics.

We performed checks for multicollinearity to ensure that explanatory variables in the regression models did not exhibit significant correlation. Marginal effects were interpreted as the absolute risk differences attributable to each factor, with all other covariates held at their mean values. We conducted data cleaning and management in SAS 9 (SAS Institute Inc, Cary, NC), while all statistical analyses were performed using Stata 18.0 (StataCorp, LLC, College Station, TX). Confidence intervals were set at the 95% level, and statistical significance was determined at *P* < .05. Robustness checks included re-estimating regression models with the composite SES score as a continuous variable and incorporating individual comorbidities measured within the ECI score framework.

## RESULTS

A total of 3,894,785 individuals were included in this analysis, of whom 74,685 (1.9%) had at least one ED visit related to a non-traumatic dental condition during the study period. Sample descriptive statistics are presented in Table 1. The majority of the sample identified as White (67.8%) and female (55.0%). The most represented age group was 35–54 years of age (29.5%). Regarding socioeconomic indicators, most individuals reported owning a home (82.5%) and were not dual-eligible for Medicare and Medicaid (85.8%). Over one-quarter of the sample reported an annual income < $40,000 (26.6%) and a net worth of < $25,000 (29.6%). Fewer than one in five individuals had attained education beyond a bachelor’s degree (17.9%). On average, individuals in the sample had 2.3 ED visits during the study period.

Bivariate relationships are presented in Table 2. Nearly all demographic characteristics were statistically different between individuals who had an ED visit for a non-traumatic dental condition compared to those who visited the ED for other reasons, with the exception of sex (*P* = .92). For instance, a higher percentage of Black individuals had an ED visit related to a non-traumatic dental condition than for other reasons (19.7% vs. 13.6%; *P* < .001). With regards to socioeconomic indicators, those who had an ED visit related to a non-traumatic dental conditions were more likely to have a high school degree or less (34.9% vs 27.5%), rent their home (26.4% vs 17.3%), income < $40,000 (38.6% vs 26.4%), net worth < $25,000 (43.1% vs 29.4%), and were on a low-income subsidy (22.1% vs 14.0%) (all *P*-values < .001). Overall, a larger proportion of individuals with a composite SES score in the lowest quartile visited the ED for a non-traumatic dental condition than for other reasons (37.6% vs 24.0%). Those who visited the ED for non-traumatic dental conditions also had a higher overall ECI score (2.4 vs 1.6).

The marginal effects of socioeconomic indicators on ED visits related to non-traumatic dental conditions, controlling for individual demographic and disease burden characteristics, are summarized in Table 3. Among the indicators assessed, income demonstrated the strongest association with ED visits for non-traumatic dental conditions, followed by homeownership status, net worth, low-income subsidy status, and education level. Compared to individuals earning > $100,000 annually, those with lower incomes showed an increased likelihood of ED visits for non-traumatic dental conditions: < $40,000 (0.7 percentage points [pp], 95% CI, 0.6–0.7); $40,000–$59,999 (0.5 pp, 95% CI, 0.4–0.6); $60,000–$74,999 (0.4 pp, 95% CI, 0.3–0.4), and $75,000–$99,999 (0.3 pp, 95% CI, 0.2–0.3). Similarly, renters were 0.4 pp (95% CI, 0.3–0.4) more likely than homeowners to visit the ED for non-traumatic dental conditions. Those with a net worth < $25,000 or receiving a low-income subsidy were also significantly more likely to have non-traumatic dental condition-related ED visits (*P* < .001). Additionally, individuals with less than a bachelor’s degree were 0.2 pp (95% CI, 0.1–0.2) more likely to use the ED for non-traumatic dental conditions compared to those with higher education. Race, sex, age, and disease burden (measured by ECI score) were also significantly associated with ED use for non-traumatic dental conditions. For example, compared to Whites, Blacks were 0.1 pp (95% CI, 0.1–0.1) more likely, whereas Hispanics were 0.4 pp (95% CI, −0.4 to −0.3) less likely, to visit the ED for a non-traumatic dental condition.

The effects of the composite SES score, adjusted for demographic and disease burden characteristics, are shown in Table 4. Compared to individuals in the highest SES quartile, those in the lowest quartile were 1.3 pp (95% CI, 1.3–1.3) more likely, those in the second quartile were 0.7 pp (95% CI, 0.6–0.7) more likely, and those in the third quartile were 0.3 pp (95% CI, 0.3–0.3) more likely to visit the ED for a non-traumatic dental condition. Marginal effects for demographic and disease burden characteristics in this model remained consistent with those in the model that accounted for each individual SES indicator.

## DISCUSSION

In this study we examined the association between individual-level SES and preventable ED use for non-traumatic dental conditions. To our knowledge, this is the first study to analyze the association between SES at the individual level and ED use for non-traumatic dental conditions. Our findings indicate that all SES factors examined were significantly related to non-traumatic dental conditions or poor oral health, with a composite SES score demonstrating a dose-dependent effect. Notably, individuals in the lowest composite SES score quartile had a 68% higher likelihood of visiting an ED for a non-traumatic dental condition compared to the general population, underscoring the profound impact of socioeconomic disparities even within a medically insured cohort. Given these results among those who have health insurance, we hypothesize that this trend would be magnified in the uninsured population, further reinforcing the need for policy interventions to address upstream social determinants of health and modifiable barriers to care.

While this is the first study to directly link individual-level SES factors to ED use for non-traumatic dental conditions, it aligns with a growing body of research demonstrating that patients with low SES have a higher likelihood of using the ED for preventable conditions. 44–46 Given that pain is a common reason individuals seek emergency care, 47,48 it is likely that non-traumatic dental conditions associated with acute pain, such as dental abscesses or severe caries, are disproportionately represented among ED visits.^15^,^49^/ Moreover, pain conditions are known to be more prevalent among lower SES individuals,^50^T raising the possibility that the observed SES disparities in ED use for non-traumatic dental conditions may be partially mediated by comorbid pain conditions. Future research should explore whether underlying pain burden contributes to disparities in ED use for non-traumatic dental conditions and whether integrated pain management strategies could mitigate the need for emergency dental care.

Among individual SES factors, income showed the strongest association with poor oral health, reinforcing the well-established link between financial hardship and barriers to accessing dental care.^51^ Our results align with previous research, including the work of Amen et al, which examined ED use for non-traumatic dental conditions in the privately insured population.^50^ Notably, while Amen et al^50^ provided foundational insights into ED use for non-traumatic dental conditions for those with private medical insurance, their study was subject to administrative data limitations that precluded an analysis of SES and race/ethnicity. In contrast, our study extends knowledge by examining ED use at the individual level while controlling for race/ethnicity and offering a more nuanced understanding of how SES influences oral health outcomes are relevant to healthcare delivery systems.

Our understanding of how individual-level SES influences ED use for non-traumatic dental conditions has been largely constrained by the fragmentation between oral and medical care delivery, which continues to limit our ability to fully explore the oral-systemic health connection and expand the evidence base in this field.^19^,^52^ Due to the structural separation of these two systems, research using secondary data that spans both domains has historically relied on administrative claims data—datasets that, while useful, lack detailed information on SES at the patient level. However, bridging the gap between dental and medical data through advanced informatics approaches, such as integrating electronic health record (EHR) data with electronic dental record data and claims data, could provide a more nuanced understanding of the relationship between SES and oral health, opening new pathways for addressing disparities and improving healthcare.^53^

Our findings, which leverage a unique administrative claims dataset linked to individual-level SES information, underscore the vast potential of EHRs, which capture more granular demographic variables, disease-related symptoms, and increasingly, health-related social needs. Recent policy efforts, such as the push by the Centers for Medicare & Medicaid Services for standardized health-related social needs data collection,^54^–^56^ further highlight the growing recognition of the need for more comprehensive data sources to inform care delivery research, particularly for ED use. By integrating these data sources with machine-learning approaches, researchers and policymakers can develop a deeper understanding of how SES factors contribute to oral health disparities, ultimately informing more effective, targeted interventions. Overall, more research is warranted, including both retrospective studies leveraging diverse datasets and prospective investigations that enable real-time enrollment and assessment of individual-level SES among patients presenting with non-emergent dental pain. Such work will be critical for generating actionable insights to guide policy, improve care delivery, and reduce persistent oral health disparities.

## LIMITATIONS

This study has several limitations that should be considered when interpreting the findings. First, because this was an observational study, we could not establish causal relationships between SES and ED use for non-traumatic dental conditions. Second, the dataset does not include information on uninsured individuals, limiting the generalizability of the findings to populations with medical insurance coverage. For instance, the proportion of homeowners in our sample is higher than the national average and likely reflects underlying differences between insured and general populations, as those with private or Medicare Advantage coverage tend to have greater financial stability and higher rates of homeownership. Thus, our findings may not generalize to broader populations not captured in the Optum database. Third, the study was constrained by Optum’s data use agreement, which prohibits linking SES data to ZIP-code-level indicators, making it impossible to account for neighborhood-level factors that may also influence poor oral health outcomes and ED use.

Additionally, the dataset lacked information on dental insurance status, a critical factor influencing access to preventive dental care. Lastly, given that we did not have granular geographic data on those included in the study, we could not account for variations in state Medicaid policies, which may significantly impact low-income beneficiaries’ access to dental care and their likelihood of seeking treatment in EDs. These limitations highlight the need for future research that integrates broader datasets and considers additional contextual factors to provide a more comprehensive understanding of the determinants of non-traumatic dental conditions-related ED use. Despite these limitations, the study has notable strengths, including its use of a large, nationally representative sample encompassing all 50 states. Furthermore, to our knowledge, this study is the first to examine individual-level SES data in relation to ED use for non-traumatic dental conditions, offering novel insights into how SES influences healthcare utilization at the individual level.

## CONCLUSION

Overall, our findings reinforce that ED visits for non-traumatic dental conditions extend beyond dental conditions alone, reflecting broader systemic barriers in health quality, efficiency, and accessibility.^57^,^58^ The association between SES and oral health disparities underscores the urgent need for policy interventions that address upstream social determinants of health. Furthermore, our work suggests that medical systems, despite the existing oral-medical divide in healthcare delivery, capture valuable health-related data that could be leveraged to better understand and mitigate oral health disparities. Integrating these insights across healthcare sectors could play a crucial role in reducing preventable ED visits and improving oral health outcomes, particularly for socioeconomically vulnerable populations.

## Supplementary Information



## Figures and Tables

**Table 1:** Sample characteristics of individuals who presented to the emergency department between 2017–2021, including demographics, socioeconomic indicators, regional distribution, and disease burden measures (N = 3,894,785).

	Frequency	Percentage
Primary outcome of interest		
Visited ED for a non-traumatic dental condition		
Yes	74,685	1.9
No	3,820,100	98.1
Demographics		
Race/Ethnicity		
Asian	121,210	3.1
Black	523,802	13.5
Hispanic	539,371	13.8
White	2,639,240	67.8
Unknown	71,162	1.8
Sex		
Female	2,142,995	55.0
Male	1,751,489	45.0
Unknown	301	<.01
Age		
18–34 years of age	798,491	20.5
35–54	1,148,110	29.5
5–64	706,009	18.1
65–74	591,906	15.2
75–84	394,840	10.1
≥ 85	255,429	6.6
Location/Census Region		
New England	143,205	3.7
Middle Atlantic	245,274	6.3
East North Central	584,403	15.0
West North Central	453,130	11.6
South Atlantic	993,210	25.5
East South Central	173,319	4.5
West South Central	628,805	16.1
Mountain	350,773	9.0
Pacific	320,268	8.2
Unknown	2,398	0.1
Socioeconomic indicators		
Education level		
High school degree or less	1,077,369	27.6
Less than a bachelor’s degree	2,121,684	54.5
Bachelor’s degree or higher	695,732	17.9
Home ownership		
Homeowner	3,214,179	82.5
Renter	680,606	17.5
Income		
< $40K	1,036,546	26.6
$40K – $49K	312,162	8.0
$50K – $59K	303,206	7.8
$60K – $74K	393,242	10.1
$75K - $99K	566,420	14.5
$100K+	1,283,209	33.0
Net worth		
< $25K	1,153,280	29.6
$25K – $149K	731,683	18.8
$150K – $249K	364,183	9.3
$250K – $499K	583,890	15.0
$500K+	1,061,749	27.3
Low-income subsidy/dual-eligible indicator		
Low-income subsidy	551,744	14.2
No low-income subsidy	3,343,041	85.8
Composite SES score (continuous)	3.69 (.92)	
Composite SES score (categorical)		
Lowest quartile	945,588	24.3
Second quartile	971,004	24.9
Third quartile	836,804	21.5
Highest quartile	1,141,389	29.3
Disease burden indicators		
Total number of ED visits (SD)	2.29 (3.08)	
Total number of years (SD)	1.42 (.78)	
Overall Elixhauser Comorbidity Index score (SD)	1.64 (2.33)	

Sample derived from a database comprising administrative health claims from a large, national, managed care company and Medicare Advantage health plan members (2017–2021).

*ED*, emergency department; *K*, thousand; *SES*, socioeconomic status.

**Table 2:** Bivariate comparisons of demographic, socioeconomic, and disease burden characteristics by whether individuals had an emergency department visit with or without a diagnosis for a non-traumatic dental condition between 2017–2021 (N = 3,894,785).

	ED visit with non-traumatic dental condition diagnosis N (%)	ED visit for other diagnoses N (%)	*P*-value
Demographic factors			
Race/Ethnicity			
Asian	1,556 (2.1)	119,654 (3.2)	< .001
Black	14,512 (19.7)	509,290 (13.6)	
Hispanic	8,860 (12.1)	530,511 (14.1)	
White	48,555 (66.1)	2,590,685 (69.1)	
Sex			
Female	41,081 (55.0)	2,101,914 (55.0)	.92
Male	33,602 (45.0)	1,717,887 (45.0)	
Age			
18–34 years of age	13,646 (18.3)	784,845 (20.6)	< .001
35–54	23,991 (32.1)	1,124,119 (29.4)	
55–64	14,857 (19.9)	691,152 (18.1)	
65–74	11,524 (15.4)	580,382 (15.2)	
75–84	7,002 (9.4)	387,838 (10.1)	
≥ 85	3,665 (4.9)	251,764 (6.6)	
Location/Census Region			
New England	2,942 (3.9)	140,263 (3.7)	< .001
Middle Atlantic	4,391 (5.9)	240,883 (6.3)	
East North Central	11,187 (15.0)	573,216 (15.0)	
West North Central	8,953 (12.0)	444,177 (11.6)	
South Atlantic	20,860 (28.0)	972,350 (25.5)	
East South Central	3,901 (5.2)	169,418 (4.4)	
West South Central	11,791 (15.8)	617,014 (16.2)	
Mountain	6,049 (8.1)	344,724 (9.0)	
Pacific	4,592 (6.1)	315,676 (8.3)	
Socioeconomic Indicators			
Education Level			
High school degree or less	26,075 (34.9)	1,051,294 (27.5)	< .001
Less than a bachelor’s degree	39,349 (52.7)	2,082,335 (54.5)	
Bachelor’s degree or higher	9,261 (12.4)	686,471 (18.0)	
Home Ownership			
Homeowner	54,976 (73.6)	3,159,203 (82.7)	< .001
Renter	19,709 (26.4)	660,897 (17.3)	
Income			
< $40K	28,056 (38.6)	1,007,683 (26.4)	< .001
$40K – $49K	6,927 (9.3)	305,235 (8.0)	
$50K – $59K	6,209 (8.3)	296,997 (7.8)	
$60K – $74K	6,995 (9.4)	386,247 (10.1)	
$75K – $99K	9,303 (12.5)	557,117 (14.6)	
$100K+	16,388 (21.9)	1,266,821 (33.1)	
Net worth			
< $25K	32,207 (43.1)	1,121,073 (29.4)	< .001
$25K – $149K	14,000 (18.8)	717,683 (18.7)	
$150K – $249K	6,152 (8.2)	358,031 (9.4)	
$250K – $499K	8,819 (11.8)	575,071 (15.1)	
$500K+	13,507 (18.1)	1,048,242 (27.4)	
Low-income subsidy/dual-eligible indicator			
Low-income subsidy	16,529 (22.1)	535,215 (14.0)	< .001
No low-income subsidy	56,156 (77.9)	3,284,885 (86.0)	
Composite SES score (continuous) (SD)	3.69 (0.92)	3.36 (0.94)	
Composite SES score (categorical)			
Lowest quartile	28,061 (37.6)	917,527 (24.0)	<. 001
Second quartile	19,453 (26.0)	951,551 (24.9)	
Third quartile	13,011 (17.4)	823,793 (21.6)	
Highest quartile	14,160 (19.0)	1,127,229 (29.5)	
Disease burden indicators			
Total number of ED visits (SD)	5.0 (8.4)	2.2 (2.9)	
Total number of years (SD)	1.9 (1.2)	1.4 (0.8)	
Overall Elixhauser Comorbidity Index score (SD)	2.4 (2.9)	1.6 (2.3)	

Source: Sample derived from a database comprising administrative health claims from a large, national, managed care company and Medicare Advantage health plan members (2017–2021).

*ED*, emergency department; *K*, thousand; *SES*, socioeconomic status.

**Table 3:** Marginal effects from a multivariable logistic regression model estimating the association between individual-level demographic, socioeconomic, and disease burden characteristics and the likelihood of an emergency department visit for a non-traumatic dental condition diagnosis (N = 3,894,785).

	ED visit with non-traumatic dental conditions diagnosis dy/dx^a^	Confidence Interval^b^	*P*-value
Demographic factors			
Race/Ethnicity			
Asian	−0.003	(−0.004, −0.002)	< .001
Black	0.001	(0.001, 0.001)	< .001
Hispanic	−0.004	(−0.004, −0.003)	< .001
White	Reference	Reference	Reference
Sex			
Female	−0.002	(−0.003, −0.002)	< .001
Male	Reference	Reference	Reference
Age			
18–34 years of age	0.010	(0.010, 0.011)	< .001
35–54	0.013	(0.013, 0.014)	< .001
55–64	0.011	(0.010, 0.012)	< .001
65–74	0.007	(0.006, 0.007)	< .001
75–84	0.003	(0.002, 0.003)	< .001
≥ 85	Reference	Reference	Reference
Geographic Location (Census Region)			
New England	Reference	Reference	Reference
Middle Atlantic	−0.002	(−0.003, −0.007)	.001
East North Central	−0.002	(−0.003, −0.001)	< .001
West North Central	−0.001	(−0.002, −0.001)	.01
South Atlantic	−0.003	(−0.004, −0.002)	< .001
East South Central	−0.003	(−0.004, −0.002)	< .001
West South Central	−0.003	(−0.004, −0.002)	< .001
Mountain	−0.003	(−0.004, −0.002)	< .001
Pacific	−0.004	(−0.005, −0.003)	< .001
Socioeconomic Indicators			
Education Level			
High school degree or less	0.002	(0.001, 0.002)	< .001
Less than a bachelor’s degree	0.002	(0.001, 0.002)	< .001
Bachelor’s degree or higher	Reference	Reference	Reference
Home Ownership			
Homeowner	Reference	Reference	Reference
Renter	0.004	(0.003, 0.004)	< .001
Income			
< $40K	0.007	(0.006, 0.007)	< .001
$40K – $49K	0.005	(0.004, 0.006)	< .001
$50K – $59K	0.005	(0.004, 0.006)	< .001
$60K – $74K	0.004	(0.003, 0.004)	< .001
$75K – $99K	0.003	(0.002, 0.003)	< .001
$100K+	Reference	Reference	Reference
Net worth			
< $25K	0.003	(0.002, 0.003)	< .001
$25K – $149K	0.001	(0.001, 0.002)	< .01
$150K – $249K	0.001	(0.001, 0.003)	.02
$250K – $499K	0.001	(−0.001, 0.007)	.59
$500K+	Reference	Reference	Reference
Low-income subsidy/dual-eligible indicator			
Low-income subsidy	0.003	(0.003, 0.004)	< .001
No low-income subsidy	Reference	Reference	Reference
Disease Burden Indicators			
Total number of ED visits	0.001	(0.001, 0.001)	< .001
Total number of years	0.008	(0.008, 0.008)	< .001
Overall Elixhauser Comorbidity Index score	0.001	(0.001, 0.001)	< .001

Source: Sample derived from a database comprising administrative health claims from a large, national, managed care company and Medicare Advantage health plan members (2017–2021).

a dy/dx reports the marginal effect: the change in the expected value of the dependent variable associated with a one-unit increase in the covariate, holding other variables constant.

b Confidence interval calculated as β ± (1.96 × standard error).

*ED*, emergency department; *K*, thousand.

**Table 4:** Marginal effects from a logistic regression model estimating the association between individual composite socioeconomic status and an emergency department visit with a non-traumatic dental condition diagnosis, adjusting for demographic, geographic, and disease burden factors (N = 3,894,785).

	ED visit with non-traumatic dental conditions diagnosis dy/dx^a^	Confidence Interval^b^	*P*-value
Demographic factors			
Race/Ethnicity			
Asian	−0.003	(−0.004, −0.002)	< .001
Black	0.001	(0.001, 0.002)	< .001
Hispanic	−0.003	(−0.004, −0.003)	< .001
White	Reference	Reference	Reference
Sex			
Female	−0.002	(−0.002, −0.002)	< .001
Male	Reference	Reference	Reference
Age			
18–34 years of age	0.010	(0.010, 0.010)	< .001
35–54	0.013	(0.012, 0.013)	< .001
55–64	0.011	(0.010, 0.011)	< .001
65–74	0.007	(0.006, 0.007)	< .001
75–84	0.003	(0.002, 0.003)	< .001
≥ 85	Reference	Reference	Reference
Geographic Location (Census Region)			
New England	Reference	Reference	Reference
Middle Atlantic	−0.002	(−0.003, −0.001)	< .001
East North Central	−0.003	(−0.004, −0.002)	< .001
West North Central	−0.002	(−0.002, −0.001)	< .001
South Atlantic	−0.003	(−0.004, −0.002)	< .001
East South Central	−0.003	(−0.004, −0.002)	< .001
West South Central	−0.004	(−0.005, −0.003)	< .001
Mountain	−0.003	(−0.004, −0.002)	< .001
Pacific	−0.004	(−0.005, −0.003)	< .001
Socioeconomic Status			
Composite SES score			
Lowest quartile	0.013	(0.013, 0.013)	< .001
Second quartile	0.007	(0.006, 0.007)	< .001
Third quartile	0.003	(0.003, 0.003)	< .001
Highest quartile	Reference	Reference	Reference
Disease Burden Indicators			
Total number of ED visits	0.001	(0.001, 0.001)	< .001
Total number of years	0.008	(0.008, 0.008)	< .001
Overall Elixhauser Comorbidity Index score	0.001	(0.001, 0.001)	< .001

Source: Sample derived from a database comprising of administrative health claims from a large, national, managed care company and Medicare Advantage health plan members (2017–2021).

a dy/dx reports the marginal effect: the change in the expected value of the dependent variable associated with a one-unit increase in the covariate, holding other variables constant.

b Confidence interval calculated as β ± (1.96 × standard error).

*ED*, emergency department; *SES*, socioeconomic status.
