# Supplementary material for: Association Between Socioeconomic Status and Emergency Department Use for Non-traumatic Dental Conditions
Source: West J Emerg Med. 2026 Jan 21;27(2):471–82. doi: 10.5811/westjem.47316 (PMC13016056; doi:10.5811/westjem.47316)
Supplement: Supplementary file 1 [file wjem-27-471-s001.docx]

**Appendix**

**ICD-10 Codes indicative of non-traumatic dental conditions**

| **ICD-10 Code** | **ICD-10 Code Description** |
| --- | --- |
| A690 | Necrotizing ulcerative stomatitis |
| A691 | Other Vincent’s infections |
| B002 | Herpesviral gingivostomatitis and pharyngotonsillitis |
| B084 | Enteroviral vesicular stomatitis with exanthem |
| B0861 | Bovine stomatitis |
| B370 | Candidal stomatitis |
| B3783 | Candidal cheilitis |
| K00 | Disorders of tooth development and eruption |
| K000 | Anodontia |
| K001 | Supernumerary teeth |
| K002 | Abnormalities of size and form of teeth |
| K003 | Mottled teeth |
| K004 | Disturbances in tooth formation |
| K005 | Hereditary disturbances in tooth structure |
| K006 | Disturbances in tooth eruption |
| K007 | Teething syndrome |
| K008 | Other disorders of tooth development |
| K009 | Disorder of tooth development, unspecified |
| K01 | Embedded and impacted teeth |
| K010 | Embedded teeth |
| K011 | Impacted teeth |
| K02 | Dental caries |
| K023 | Arrested dental caries |
| K025 | Dental caries on pit and fissure surface |
| K0251 | Dental caries on pit and fissure surface limited to enamel |
| K0252 | Dental caries on pit and fissure surface penetrat into dentin |
| K0253 | Dental caries on pit and fissure surface penetrat into pulp |
| K026 | Dental caries on smooth surface |
| K0261 | Dental caries on smooth surface limited to enamel |
| K0262 | Dental caries on smooth surface penetrating into dentin |
| K0263 | Dental caries on smooth surface penetrating into pulp |
| K027 | Dental root caries |
| K029 | Dental caries, unspecified |
| K03 | Other diseases of hard tissues of teeth |
| K030 | Excessive attrition of teeth |
| K031 | Abrasion of teeth |
| K032 | Erosion of teeth |
| K033 | Pathological resorption of teeth |
| K034 | Hypercementosis |
| K035 | Ankylosis of teeth |
| K036 | Deposits [accretions] on teeth |
| K037 | Posteruptive color changes of dental hard tissues |
| K038 | Other specified diseases of hard tissues of teeth |
| K0381 | Cracked tooth |
| K0389 | Other specified diseases of hard tissues of teeth |
| K039 | Disease of hard tissues of teeth, unspecified |
| K04 | Diseases of pulp and periapical tissues |
| K040 | Pulpitis |
| K0401 | Reversible pulpitis |
| K0402 | Irreversible pulpitis |
| K041 | Necrosis of pulp |
| K042 | Pulp degeneration |
| K043 | Abnormal hard tissue formation in pulp |
| K044 | Acute apical periodontitis of pulpal origin |
| K045 | Chronic apical periodontitis |
| K046 | Periapical abscess with sinus |
| K047 | Periapical abscess without sinus |
| K048 | Radicular cyst |
| K049 | Other and unspecified diseases of pulp and periapical tissues |
| K0490 | Unspecified diseases of pulp and periapical tissues |
| K0499 | Other diseases of pulp and periapical tissues |
| K05 | Gingivitis and periodontal diseases |
| K050 | Acute gingivitis |
| K0500 | Acute gingivitis, plaque induced |
| K0501 | Acute gingivitis, non-plaque induced |
| K051 | Chronic gingivitis |
| K0510 | Chronic gingivitis, plaque induced |
| K0511 | Chronic gingivitis, non-plaque induced |
| K052 | Aggressive periodontitis |
| K0520 | Aggressive periodontitis, unspecified |
| K0521 | Aggressive periodontitis, localized |
| K05211 | Aggressive periodontitis, localized, slight |
| K05212 | Aggressive periodontitis, localized, moderate |
| K05213 | Aggressive periodontitis, localized, severe |
| K05219 | Aggressive periodontitis, localized, unspecified severity |
| K0522 | Aggressive periodontitis, generalized |
| K05221 | Aggressive periodontitis, generalized, slight |
| K05222 | Aggressive periodontitis, generalized, moderate |
| K05223 | Aggressive periodontitis, generalized, severe |
| K05229 | Aggressive periodontitis, generalized, unspecified severity |
| K053 | Chronic periodontitis |
| K0530 | Chronic periodontitis, unspecified |
| K0531 | Chronic periodontitis, localized |
| K05311 | Chronic periodontitis, localized, slight |
| K05312 | Chronic periodontitis, localized, moderate |
| K05313 | Chronic periodontitis, localized, severe |
| K05319 | Chronic periodontitis, localized, unspecified severity |
| K0532 | Chronic periodontitis, generalized |
| K05321 | Chronic periodontitis, generalized, slight |
| K05322 | Chronic periodontitis, generalized, moderate |
| K05323 | Chronic periodontitis, generalized, severe |
| K05329 | Chronic periodontitis, generalized, unspecified severity |
| K054 | Periodontitis |
| K0540 |  |
| K055 | Other periodontal diseases |
| K056 | Periodontal disease, unspecified |
| K06 | Other disorders of gingiva and edentulous alveolar ridge |
| K060 | Gingival recession |
| K0601 | Gingival recession, localized |
| K06010 | Localized gingival recession, unspecified |
| K06011 | Localized gingival recession, minimal |
| K06012 | Localized gingival recession, moderate |
| K06013 | Localized gingival recession, severe |
| K0602 | Gingival recession, generalized |
| K06020 | Generalized gingival recession, unspecified |
| K06021 | Generalized gingival recession, minimal |
| K06022 | Generalized gingival recession, moderate |
| K06023 | Generalized gingival recession, severe |
| K061 | Gingival enlargement |
| K063 | Horizontal alveolar bone loss |
| K068 | Other specified disorders of gingiva and edentulous alveolar ridge |
| K069 | Disorder of gingiva and edentulous alveolar ridge, unspecified |
| K08 | Other disorders of teeth and supporting structures |
| K080 | Exfoliation of teeth due to systemic causes |
| K081 | Complete loss of teeth |
| K0810 | Complete loss of teeth, unspecified cause |
| K08101 | Complete loss of teeth, unspecified cause, class I |
| K08102 | Complete loss of teeth, unspecified cause, class II |
| K08103 | Complete loss of teeth, unspecified cause, class III |
| K08104 | Complete loss of teeth, unspecified cause, class IV |
| K08109 | Complete loss of teeth, unspecified cause, unspecified class |
| K0812 | Complete loss of teeth due to periodontal diseases |
| K08121 | Complete loss of teeth due to periodontal disease, class I |
| K08122 | Complete loss of teeth due to periodontal disease, class II |
| K08123 | Complete loss of teeth due to periodontal disease, class III |
| K08124 | Complete loss of teeth due to periodontal disease, class IV |
| K08129 | Complete loss of teeth due to periodontal disease, unspecified class |
| K0813 | Complete loss of teeth due to caries |
| K08131 | Complete loss of teeth due to caries, class I |
| K08132 | Complete loss of teeth due to caries, class II |
| K08133 | Complete loss of teeth due to caries, class III |
| K08134 | Complete loss of teeth due to caries, class IV |
| K08139 | Complete loss of teeth due to caries, unspecified class |
| K0819 | Complete loss of teeth due to other specified cause |
| K08191 | Complete loss of teeth due to other specified cause, class I |
| K08192 | Complete loss of teeth due to other specified cause, class II |
| K08193 | Complete loss of teeth due to other specified cause, class III |
| K08194 | Complete loss of teeth due to other specified cause, class IV |
| K08199 | Complete loss of teeth due to other specified cause, unspecified class |
| K082 | Atrophy of edentulous alveolar ridge |
| K0820 | Unspecified atrophy of edentulous alveolar ridge |
| K0821 | Minimal atrophy of the mandible |
| K0822 | Moderate atrophy of the mandible |
| K0823 | Severe atrophy of the mandible |
| K0824 | Minimal atrophy of maxilla |
| K0825 | Moderate atrophy of the maxilla |
| K0826 | Severe atrophy of the maxilla |
| K083 | Retained dental root |
| K084 | Partial loss of teeth |
| K0840 | Partial loss of teeth, unspecified cause |
| K08401 | Partial loss of teeth, unspecified, class I |
| K08402 | Partial loss of teeth, unspecified, class II |
| K08403 | Partial loss of teeth, unspecified, class II |
| K08404 | Partial loss of teeth, unspecified, class IV |
| K08409 | Partial loss of teeth, unspecified cause, unspecified class |
| K0842 | Partial loss of teeth due to periodontal diseases |
| K08421 | Partial loss of teeth due to periodontal diseases, class I |
| K08422 | Partial loss of teeth due to periodontal diseases, class II |
| K08423 | Partial loss of teeth due to periodontal diseases, class III |
| K08424 | Partial loss of teeth due to periodontal diseases, class IV |
| K08429 | Partial loss of teeth due to periodontal disease, unspecified class |
| K0843 | Partial loss of teeth due to caries |
| K08431 | Partial loss of teeth due to caries, class I |
| K08432 | Partial loss of teeth due to caries, class II |
| K08433 | Partial loss of teeth due to caries, class II |
| K08434 | Partial loss of teeth due to caries, class III |
| K08439 | Partial loss of teeth due to caries, unspecified class |
| K0849 | Partial loss of teeth due to other specified cause |
| K08491 | Partial loss of teeth due to other specified cause, class I |
| K08492 | Partial loss of teeth due to other specified cause, class II |
| K08493 | Partial loss of teeth due to other specified cause, class III |
| K08494 | Partial loss of teeth due to other specified cause, class IV |
| K08499 | Partial loss of teeth due to other cause, unspecified class |
| K0850 | Unsatisfactory restoration of tooth, unspecified |
| K0851 | Open restoration margins of tooth |
| K0852 | Unrepairable overhanging of dental restorative materials |
| K0853 | Fractured dental restorative material |
| K08530 | Fractured dental restorative material without loss of material |
| K08531 | Fractured dental restorative material with loss of material |
| K08539 | Fracture dental restorative material, unspecified |
| K0854 | Contour of existing restoration of tooth biologically incompatible with oral health |
| K0855 | Allergy to existing dental restorative material |
| K0856 | Poor aesthetic of existing restoration of tooth |
| K0859 | Other unsatisfactory restoration of tooth |
| K088 | Other specified disorders of teeth and supporting structures |
| K0889 | Other specified disorders of teeth and supporting structures |
| K089 | Disorder of teeth and supporting structures, unspecified |
| K09 | Cysts of oral region, not elsewhere classified |
| K090 | Developmental odontogenic cysts |
| K091 | Developmental (nonodontogenic) cysts of oral region |
| K098 | Other cyst of oral region, not elsewhere classified |
| K099 | Cyst of oral region, unspecified |
| K11 | Diseases of salivary glands |
| K110 | Atrophy of salivary gland |
| K111 | Hypertrophy of salivary gland |
| K112 | Sialoadenitis |
| K1120 | Sialodenitis, unspecified |
| K1121 | Acute sialoadenitis |
| K1122 | Acute recurrent sialoadenitis |
| K1123 | Chronic sialoadenitis |
| K113 | Abscess of salivary gland |
| K114 | Fistula of salivary gland |
| K115 | Sialolithiasis |
| K116 | Mucocele of salivary gland |
| K117 | Disturbances of salivary secretion |
| K118 | Other diseases of salivary glands |
| K119 | Disease of salivary gland, unspecified |
| K12 | Stomatitis and related lesions |
| K120 | Recurrent oral aphthae |
| K121 | Other forms of stomatitis |
| K122 | Cellulitis and abscess of mouth |
| K123 | Oral mucositis (ulcerative) |
| K1230 | Oral mucositis (ulcerative), unspecified |
| K1231 | Oral mucositis (ulcerative), due to antineoplastic therapy |
| K1232 | Oral mucositis (ulcerative), due to other drugs |
| K1233 | Oral mucositis (ulcerative), due to radiation |
| K1239 | Other oral mucositis (ulcerative) |
| K13 | Other diseases of lip and oral mucosa |
| K130 | Disease of lips |
| K131 | Cheek and lip biting |
| K132 | Leukoplakia and other disturbances of oral epithelium, including tongue |
| K1321 | Leukoplakia and other disturbances of oral epithelium, including tongue |
| K1322 | Minimal keratinized residual ridge mucosa |
| K1323 | Excessive keratinized residual ridge mucosa |
| K1324 | Leukokeratosis nicotina palati |
| K1329 | Other disturbances of oral epithelium, including tongue |
| K133 | Hairy leukoplakia |
| K134 | Granuloma and granuloma-like lesions of oral mucosa |
| K135 | Oral submucous fibrosis |
| K136 | Irritative hyperplasia of oral mucosa |
| K137 | Other and unspecified lesions of oral mucosa |
| K1370 | Unspecified lesions of oral mucosa |
| K1379 | Other lesions of oral mucosa |
| K14 | Glossitis |
| K140 | Glossitis |
| K141 | Geographic tongue |
| K142 | Median rhomboid glossitis |
| K143 | Hypertrophy of tongue papillae |
| K144 | Atrophy of tongue papillae |
| K145 | Plicated tongue |
| K146 | Glossodynia |
| K148 | Other diseases of tongue |
| K149 | Disease of tongue, unspecified |
| M26 | Dentofacial anomalies [including malocclusion] |
| M260 | Major anomalies of jaw size |
| M2600 | Unspecified anomaly of jaw size |
| M2601 | Maxillary hyperplasia |
| M2602 | Maxillary hypoplasia |
| M2603 | Mandibular hyperplasia |
| M2604 | Mandibular hypoplasia |
| M2605 | Macrogenia |
| M2606 | Microgenia |
| M2607 | Excessive tuberosity of jaw |
| M2609 | Other specified anomalies of jaw size |
| M261 | Anomalies of jaw-cranial base relationship |
| M2610 | Unspecified anomaly of jaw-cranial base relationship |
| M2611 | Maxillary asymmetry |
| M2612 | Other jaw asymmetry |
| M2619 | Other specified anomalies of jaw-cranial base relationship |
| M262 | Anomalies of dental arch relationship |
| M2620 | Unspecified anomaly of dental arch relationship |
| M2621 | Malocclusion, Angle's class |
| M26211 | Malocclusion, Angle's class I |
| M26212 | Malocclusion, Angle's class II |
| M26213 | Malocclusion, Angle's class III |
| M26219 | Malocclusion, Angle's class unspecified |
| M2622 | Open occlusal relationship |
| M26220 | Open anterior occlusal relationship |
| M26221 | Open posterior occlusal relationship |
| M2623 | Excessive horizontal overlap |
| M2624 | Reverse articulation |
| M2625 | Anomalies of interarch distance |
| M2629 | Other anomalies of dental arch relationship |
| M263 | Anomalies of tooth position of fully erupted tooth or teeth |
| M2630 | Unspecified anomaly of tooth position of fully erupted tooth/teeth |
| M2631 | Crowding of fully erupted teeth |
| M2632 | Excessive spacing of fully erupted teeth |
| M2633 | Horizontal displacement of fully erupted tooth or teeth |
| M2634 | Vertical displacement of fully erupted tooth or teeth |
| M2635 | Rotation of fully erupted tooth or teeth |
| M2636 | Insufficient interocclusal distance of fully erupted teeth |
| M2637 | Excessive interocclusal distance of fully erupted teeth |
| M2639 | Other anomalies of tooth position of fully erupted tooth/teeth |
| M264 | Malocclusion, unspecified |
| M265 | Dentofacial functional abnormalities |
| M2650 | Dentofacial function abnormalities, unspecified |
| M2651 | Abnormal jaw closure |
| M2652 | Limited mandibular range of motion |
| M2653 | Deviation in opening and closing of the mandible |
| M2654 | Insufficient anterior guidance |
| M2655 | Centric occlusion maximum intercuspation discrepancy |
| M2656 | Non-working side interference |
| M2657 | Lack of posterior occlusal support |
| M2659 | Other dentofacial functional abnormalities |
| M266 | Temporomandibular joint disorders |
| M2660 | Temporomandibular joint disorder, unspecified |
| M26601 | Right temporomandibular joint disorder, unspecified |
| M26602 | Left temporomandibular joint disorder, unspecified |
| M26603 | Bilateral temporomandibular joint disorder, unspecified |
| M26609 | Unspecified TMJ joint disorder, unspecified side |
| M2661 | Adhesions and ankylosis of temporomandibular joint |
| M26611 | Adhesions and ankylosis of right temporomandibular joint |
| M26612 | Adhesions and ankylosis of left temporomandibular joint |
| M26613 | Adhesions and ankylosis of bilateral temporomandibular joint |
| M26619 | Adhesions and ankylosis of TMJ joint, unspecified side |
| M2662 | Arthralgia of temporomandibular joint |
| M26621 | Arthralgia of right temporomandibular joint |
| M26622 | Arthralgia of left temporomandibular joint |
| M26623 | Arthralgia of bilateral temporomandibular joint |
| M26629 | Arthralgia of temporomandibular joint, unspecified side |
| M2663 | Articular disc disorder of temporomandibular joint |
| M26631 | Articular disc disorder of right temporomandibular joint |
| M26632 | Articular disc disorder of left temporomandibular joint |
| M26633 | Articular disc disorder of bilateral temporomandibular joint |
| ICD- 10 Code | **ICD-10 Code Description** |
| M26639 | Articular disc disorder of TMJ joint, unspecified side |
| M2664 | Arthritis of temporomandibular joint |
| M26641 | Arthritis of right temporomandibular joint |
| M26642 | Arthritis of left temporomandibular joint |
| M26643 | Arthritis of bilateral temporomandibular joint |
| M26649 | Arthritis of unspecified temporomandibular joint |
| M2665 | Arthropathy of temporomandibular joint |
| M26651 | Arthropathy of right temporomandibular joint |
| M26652 | Arthropathy of left temporomandibular joint |
| M26653 | Arthropathy of bilateral temporomandibular joint |
| M26659 | Arthropathy of unspecified temporomandibular joint |
| M2669 | Other specified disorders of temporomandibular joint |
| M267 | Dental alveolar anomalies |
| M2670 | Unspecified alveolar anomaly |
| M2671 | Alveolar maxillary hyperplasia |
| M2672 | Alveolar mandibular hyperplasia |
| M2673 | Alveolar maxillary hypoplasia |
| M2674 | Alveolar mandibular hypoplasia |
| M2679 | Other specified alveolar anomalies |
| M268 | Other dentofacial anomalies |
| M2681 | Anterior soft tissue impingement |
| M2682 | Posterior soft tissue impingement |
| M2689 | Other dentofacial anomalies |
| M269 | Dentofacial anomaly, unspecified |
| M27 | Other diseases of jaws |
| M270 | Developmental disorders of jaws |
| M271 | Giant cell granuloma, central |
| M272 | Inflammatory conditions of jaws |
| M273 | Alveolitis of jaws |
| M274 | Other and unspecified cysts of jaw |
| M2740 | Unspecified cyst of jaw |
| M2749 | Other cyst s of jaw |
| M275 | Periradicular pathology associated with previous endodontic treatment |
| M2751 | Perforation of root canal space due to endodontic treatment |
| M2752 | Endodontic overfill |
| M2753 | Endodontic underfill |
| M2759 | Other periradicular pathology associated with preventive endodontic treatment |
| M276 | Endosseous dental implant failure |
| M2761 | Osseointegration failure of dental implant |
| M2762 | Post-osseointegration of biological failure of dental implant |
| M2763 | Post-osseointegration mechanical failure of dental implant |
| M2769 | Other endosseous dental implant failure |
| M278 | Other specified diseases of jaws |
| M279 | Disease of jaws, unspecified |
| M350C | Sjogren syndrome with dental involvement |
| M7911 | Myalgia of mastication muscle |
| R682 | Dry mouth, unspecified |
| R6884 | Jaw pain |
